# Identification of *Golovinomyces artemisiae* Causing Powdery Mildew, Changes in Chlorophyll Fluorescence Parameters, and Antioxidant Levels in *Artemisia selengensis*

**DOI:** 10.3389/fpls.2022.876050

**Published:** 2022-05-26

**Authors:** Zhixin Guo, Xiaoyang Sun, Ligang Qin, Lili Dong, Liangbing Xiong, Fuchun Xie, Dong Qin, Yajun Chen

**Affiliations:** ^1^College of Horticulture, Northeast Agricultural University, Harbin, China; ^2^College of Animal Science and Technology, Northeast Agricultural University, Harbin, China

**Keywords:** *Artemisia selengensis*, powdery mildew, *Golovinomyces artemisiae*, chlorophyll fluorescence, antioxidant

## Abstract

*Artemisia selengensis* Turcz. is a valuable edible and medicinal vegetable crop widely cultivated in Northeast China. Powdery mildew (PM) disease occurs during field and greenhouse cultivation, resulting in production losses and quality deterioration. The pathogen in *A. selengensis* was *Golovinomyces artemisiae* identified using optical microscopic and scanning electron microscopic observations, morphological identification, and molecular biological analyses. Parameters of chlorophyll fluorescence (ChlF) and antioxidant system responses as well as callose and lignin contents in *A. selengensis* were analyzed with inoculating *G. artemisiae*. Obvious of PM-infected leaves were confirmed with significantly lower values in electron transport rate (ETR), non-photochemical quenching (NPQ), photochemical quenching (qP), and actual photochemical efficiency [Y(II)], but higher values in non-adjusting energy dissipation yield [Y(NO)], supposed that maximal photosystem II quantum yield (Fv/Fm) value and images could be used to monitor PM degree on infected*A. selengensis*. In addition, malondialdehyde (MDA), superoxide anion (O_2_^–^), callose, lignin contents, and peroxidase (POD) activity increased, while superoxide dismutase (SOD) activity, catalase (CAT) activity, and ascorbic acid (AsA) content decreased significantly in infected leaves compared to mock-inoculated leaves, indicated that lignin and protective enzymes are the key indicators for detecting PM resistant in *A. selengensis*. These results suggest that PM caused by *G. artemisiae* disrupted the photosynthetic capacity and induced imbalance of antioxidant system in*A. selengensis*. The findings were of great significance for designing a feasible approach to effectively prevent and control the PM disease in *A. selengensis* as well as in other vegetable crops.

## Introduction

*Artemisia selengensis* Turcz. is a perennial plant belonging to the genus *Artemisia* of the Asteraceae family (Wen et al., 2016). Due to its high nutritional and medicinal value, *A. selengensis* has been favored as both a kind of vegetable and a herbal medicine in Northeast China for thousands of years (Peng et al., 2009; Wen et al., 2016). However, leaves as the main edible parts of the plant are extremely vulnerable to powdery mildew (PM) disease when the plant is cultivated in field and/or in greenhouse, especially under low air flow and high relative humidity environment in summer and autumn. This has a negative economic impact on the plant production and the overall agricultural industry. Even though PM symptoms can be easily recognized, it is challenging to determine the species assignment (Glawe, 2008). Morphological characteristics and observation of pathogen are crucial for the identification of pathogen at species and prevention of PM. For example, *Blumeria graminis* (DC.) Speer is unique in forming conidia compared to other species of *Erysiphales* (Glawe, 2008). Previous studies revealed that the main types of PM pathogens parasitizing *Asteraceae* are *Golovinomyces cichoracearum*, *Golovinomyces chrysanthemi*, and *Golovinomyces artemisiae* (Matsuda and Takamatsu, 2003; Lebeda et al., 2012; Bradshaw et al., 2017). *G. artemisiae* is described in Europe with *Artemisia vulgaris* being a type of host, of which a detailed description has been published by Braun (1995). *G. artemisiae* in *Artemisia annua* is also reported and identified using a combination of morphological and internal transcribed spacer (ITS) methods in Korea (Choi et al., 2014). However, the species of pathogen causing PM in *A. selengensis* remains unclear and phenotypic and physiological changes of *A. selengensis* plants induced by PM are rarely reported in Northeast China (Lebeda et al., 2020).

When plants are infected with PM, photosynthesis is reduced through a lower supply of light energy because of the leaf surface covered by mycelium (Scott et al., 1996). On the other hand, CO_2_ influx is inhibited due to stomata closure (Duniway, 1982; Berger et al., 2007). Previous studies have demonstrated that Erysiphe alphitoides leading to the reduction of foliage photosynthetic activity in pedunculate oak (*Quercus robur*) (Copolovici et al., 2014). Modern chlorophyll fluorescence (ChlF) technology allows the rapid and non-destructive detection of photosynthetic activity (Kuckenberg et al., 2008). Maximal photosystem II quantum yield (Fv/Fm) was used to diagnose several diseases, including coffee (*Coffea arabica* L.) infected by *Hemileia vastatrix* and cedar (*Cedrus deodara*) infected by *Pestalotiopsis* spp. (Ning et al., 1995; Honorato Júnior et al., 2015). Meanwhile, the parameter Fv/Fm could distinguish resistant and susceptible lettuce (*Lactuca sativa* L.) lines against the *Bremia lactucae* (Bauriegel et al., 2014). In terms of Fv/Fm and effective quantum yield of PSII [Y(II)], leaves infected by *Bipolaris sorokiniana* were also measured dramatically impaired on the most susceptible cultivar compared to a less susceptible cultivar in wheat (*Triticum aestivum* L.) (Rios et al., 2017). Reductions in values of Fv/Fm, Y(II), quantum yield of non-regulated energy dissipation [Y(NO)], and photochemical quenching (qP) coefficient are noticeable on necrotic vein tissues induced by *Colletotrichum truncatum* in contrast to the surrounding leaf tissue in soybean (*Glycine max* L.) (Dias et al., 2018). The non-photochemical quenching (NPQ) processes increase in *Podosphaera xanthii*-infected melon leaves, which constitute a major mechanism for the avoidance of photodamage (Polonio et al., 2019). Furthermore, different fungi have been shown to inhibit photosynthetic electron transfer reactions variably, which are a source of reactive oxygen species (ROS) (Duniway, 1982; Tang et al., 1996; Zhao et al., 2011). Lignin and callose activate the host defense system, giving the host plant time to initiate subsequent defense responses, such as ROS burst and antioxidant enzyme activity regulation (Jacobs et al., 2003; Blumke et al., 2014). Callose was accumulated in Arabidopsis (*Arabidopsis thaliana* L.) infected with PM, which enhanced its resistance to host (Ellinger et al., 2013). Meanwhile, lignin content was increased to prevent pathogens infection and spread of wheat against PM by causing cell wall suberization (Bhuiyan et al., 2009). Moreover, the increasing of lignin content can significantly improve peroxidase (POD) activity (Lee et al., 2018). To response *Glomerella cingulata* attack, POD activity was maintained at a higher level, superoxide dismutase (SOD) and catalase (CAT) were inhibited, reducing ROS scavenging capacity in susceptible cultivar compared to that of the resistance cultivar in apple (*Malus pumila*) (Zhang et al., 2016). Excess ROS would cause serious damage to plant protein and membrane system. The scavenging of O_2_^–^ depends on the high activities of SOD, POD, and CAT enzymes for rice (*Oryza sativa* L.) to resist *Magnaporthe oryzae* infection (Groß et al., 2013; Abdul et al., 2018). Malondialdehyde (MDA) increases twofold in wheat seedlings infected by *Fusarium pseudograminearum*, which has long been used as a marker of stress tolerance to lipid peroxidation (Boamah et al., 2021). Ascorbic acid (AsA), as the most abundant antioxidant in plant, can directly mitigate the damaging effects of ROS or indirectly as a substrate for the ascorbate peroxidase enzyme (Macknight et al., 2017). AsA deficiency has been found to positively modulate plant’s biotic defense cascades leading to better disease resistance response in Arabidopsis to *Pseudomonas syringae* (Pavet et al., 2005). In this scenario, the antioxidant systems exhibit an ever-increasing importance in the complex process of defense mechanisms against PM in *A. selengensis*. Nevertheless, detailed study is lacking on these indicators as regulatory mechanisms markers in *A. selengensis* infected by PM.

In this study, *G. artemisiae* was characterized using light microscopic and scanning electron microscopic (SEM) observations to investigate the responses of *A. selengensis* to PM. ITS and 28S ribosomal DNA (rDNA) regions were sequenced for supporting the identification of pathogen. We further determined the physiological and biochemical indicators such as ChlF, lignin, callose, and antioxidant enzymes in *A. selengensis* leaves infected by *G. artemisiae.* This study is a pilot study for providing basic knowledge and information for improving PM resistance of *A. selengensis* and also for other plant species.

## Materials and Methods

### Plant Materials and Powdery Mildew Isolation

*Artemisia selengensis* Turcz. was cultivated in farm field of Northeast Agricultural University, China (45°43′55′′N, 126°43′21′′E). Leaves of *A. selengensis* with typical PM colonies were sampled in September 2021, which were further used for isolating pathogen and inoculation to young seedlings. Seedlings were prepared by sowing seeds for pot culture in greenhouse. Briefly, 10 seeds of *A*. *selengensis* were sown in PVC pots with sterile substrate soil for a total of 10 pots in the early August. After the seedlings reached to 15 cm in height (nearly 40 days cultivation), the pathogen inoculation was performed. The individual isolate, which obtained from the farm leaves, was purified by single-colony inoculation on healthy seedlings for five consecutive generations (Wen et al., 2011; Lebeda et al., 2012; Rallos et al., 2016). Controlled growth conditions in greenhouse were set at 20/18°C (day/night) and 12 h of light (125 μmol m^–2^ s^–1^).

### Morphological Characterization of *Golovinomyces artemisiae*

Chasmothecia and conidia were removed from *G. artemisiae*-infected leaves with a dissecting needle, mounted in water, and observed under optical microscope (Carl Zeiss Model Axioskop 40). Taxonomic characters were examined and recorded, including chasmothecial appendages, number of asci and ascospores, and lengths and widths of conidia and conidiophore foot cells. Fifty or more measurements were made for individual characters from each sample and compared to the species pathogen descriptions by Choi et al. (2014).

### Scanning Electron Microscope Observation of *Golovinomyces artemisiae*

Leaves infected with *G. artemisiae* were cut into small squares sized 5 mm in length around veins, immediately put in a vial containing 2.5% glutaraldehyde, and fixed with 2 ml of 0.1 mol l^–1^ phosphate buffer (pH 6.8) for 3 times, 10 min each time. The leaves were gradually dehydrated using 2 ml of 50, 70, and 90% ethanol solutions for 15 min each, respectively. Leaves were transferred to a pure tert-butanol solution and let stand for 20 min and then washed with an equal volume of anhydrous ethanol and tert-butanol once and pure tert-butanol twice, with submergence for 15 min each time. Finally, the samples were put in a freezer at −20°C for 30 min and transferred into the ES-2030 (HITACHI) freeze dryer for 4 h. Afterward, ice crystals were evaporated and dried in vials and sputtered on a gold-plated film in ion coater, which were then observed and imaged by SEM (Hitachi SU-8010, Tokyo, Japan).

### Molecular Identification and Phylogenetic Analyses of *Golovinomyces artemisiae*

Total genomic DNA was isolated from 100 mg of PM (conidia and mycelia) using the cetyltrimethylammonium bromide (CTAB) method (Johanson et al., 1994). The sequence of ITS and 28S rDNA was amplified using the ITS1/ITS4 (ITS1: 5′-TCCGTAGGTGAACCTGCGG-3′, ITS4: 5′-TCCTCCGCTTATTGATATGC-3′) and PM3/TW14 (PM3: 5′-GKGCTYTMCGCGTAGT-3′, TW14: 5′-GCTATCCT GAGGGAAACTTC-3′) primers pair, respectively (White et al., 1990; Mori et al., 2000). The reaction procedure was 94°C for 10 min; 32 cycles (94°C for 30 s, 57°C for 30 s, and 72°C for 90 s); 72°C for 5 min; and 4°C termination. PCR product was purified and ligated to the pEASY-Blunt Zero vector and transformed into *Escherichia coli* and a positive strain was sequenced. The sequences were uploaded to the National Center for Biotechnology Information (NCBI) database and used as queries in BLAST^1^ searches to identify the most similar sequences available in the GenBank.

These sequences were collected and aligned for constructing the phylogenetic tree using ClustalW (Thompson et al., 1994). The maximum likelihood (ML) method was used to generate phylogenetic trees based on tandem sequences of the ITS and 28S rDNA genes using the MEGA version 7.0 software (Kumar et al., 2016). Bootstrap analysis was made using 1,000 replications (Joseph, 1985).

### Pathogenicity Assays of *Golovinomyces artemisiae*

Pathogenicity was verified by inoculating 10 healthy seedlings using the above purified PM pathogen. Different paint brushes were used to dust conidia from one PM patch onto another plant leaves of *A*. *selengensis* (Attanayake et al., 2010). Mock-inoculated (CK) leaves (i.e., no conidia were attached to the leaf surface) were used as controls to monitor and minimize potential contamination. Leaf symptoms were recorded every 1–2 days. Diseased leaves were collected for microscopic examinations to observe the morphological characteristics of the inoculated pathogens. After 14 days, *G. artemisiae* inoculation (GI) and CK leaves were used to measure the ChlF and collected immediately stored at −80°C for the determination of antioxidant-related indexes.

### Leaf Chlorophyll Fluorescence

Chlorophyll fluorescence parameters of GI and CK were measured using the Imagining-PAM (MAXI) system (Walz, Germany). The value (Ft) of the selected sample in area of interest (AOI) was set within the range of 0.1–0.2, plant saturation pulse light frequency was set to 20 s/times and the intensity was set to 4,000 μmol m^–2^ s^–1^, and the light intensity for actinic light parameters was set to 86 μmol m^–2^ s^–1^ (Shi et al., 2020). The plant samples were treated in darkness for 20 min; minimum fluorescence (Fo) and maximum fluorescence (Fm) of the samples were obtained using the measuring light and saturated pulsed light, respectively. The values and images of NPQ, actual photochemical efficiency [Y(II)], non-adjusting energy dissipation yield [Y(NO)], qP, and electron transport rate (ETR) were then obtained through actinic light measurements. Fv/Fm was calculated as: Fv/Fm = (Fm − Fo) / Fm (Maxwell and Johnson, 2000).

### Determination of Callose, Lignin, and Antioxidant-Related Indexes

For the assay of antioxidant-related index, 0.5 g of fresh leaves was homogenized using 2 ml of 50 mM phosphate extraction buffer [phosphate-buffered saline (PBS), pH 7.8] in ice-cold mortar. The mixture was centrifuged at 12,000 *g* for 15 min at 4°C for collecting the supernatant. The supernatant was used to determine the content of superoxide anion (O_2_^–^) and activities of CAT, POD, callose, and SOD. Callose contents were measured following the method of Khle et al. (1985). A total of 0.2 ml of the supernatant was put into a 1.5-ml centrifuge tube. A total of 0.4 ml aniline blue (0.1%), 0.21 ml HCl (1 mol⋅l^–1^), and 0.59 ml glycine/NaOH buffer (1 mol⋅l^–1^, pH 9.5) were added in turn, reacted at 50°C for 20 min. The mixture was cooled to room temperature and measured the fluorescence intensity with fluorescence spectrophotometer. The excitation wavelength of the measurement was 400 nm, the emission wavelength was 500 nm, and the slit width was 5 nm.

Superoxide dismutase was measured using the nitroblue tetrazolium (NBT) reduction methods (Fridovich, 1971). The reaction mixtures contained 0.3 ml of 50 mM Na carbonate (pH 10.2), 0.3 ml of 1.3 mM riboflavin, 0.3 ml of 13 mM methionine, 0.3 ml of 75 mM NBT, and 0.1 ml of supernatant. The absorbance was measured at 560 nm using a spectrophotometer (UV-2450).

Peroxidase was determined spectrophotometrically by monitoring the formation of tetraguaiacol from guaiacol (extinction coefficient at 470 nm) in the presence of hydrogen peroxide (H_2_O_2_) (Ranieri et al., 2000). The reaction mixtures consisted of 2.9 ml of 50 mM PBS (pH 7.0), 1 ml of 0.3 mM guaiacol, 1 ml of 0.1 mM hydrogen peroxide, and 0.1 ml of supernatant.

Catalase was estimated by the rate of H_2_O_2_ decomposition at 240 nm (Havir and McHale, 1989). The reaction mixture contained 0.2 ml of supernatant, 1.5 ml of PBS (PH 7.8), 1 ml of distilled water, and 0.3 ml of 100 mM H_2_O_2_. The absorbance was recorded every 1 min for a total of 4 min.

Superoxide anion content was determined from oxidation of hydroxylamine (Zhou et al., 2004). A total of 0.1 ml of supernatant was incubated at 25°C for 20 min with a mixture of 0.9 ml of 65 mM phosphate buffer (pH 7.8) and 0.1 ml of 10 mM hydroxylammonium chloride; 0.2 ml of 17 mM sulfanilamide and 0.2 ml of 7 mM α-naphthylamine were then added to the mixture and incubated again at 25°C for 20 min. An equal volume of chloroform was added. The mixture was centrifuged at 10,000 *g* for 3 min and absorbance was read at 530 nm.

Referring to the method of Morrison (1972), the lignin content was determined. A total of 0.5 g fresh leaves were ground to a homogenate by adding 95% ethanol in a mortar and the precipitate was collected after centrifugation at 4,500 rpm for 10 min. The pellet was washed three times with an equal volume of a 1:1 95% ethanol and n-hexane solution and precipitate was collected and dried. The dried product was dissolved in 0.5 ml of 25% glacial acetic acid and then set in a water bath at 70°C for 30 min. Thereafter, 0.9 ml of 2 mol/l NaOH was added to terminate the reaction. A total of 5 ml of glacial acetic acid and 0.1 ml of 7.5 mol/l hydroxylamine hydrochloride were added into mixture. After mixing and centrifugation of the samples at 4,500 rpm for 5 min, 0.1 ml of the supernatant was aspirated and diluted, with 3.0 ml of glacial acetic acid. Absorbance was measured at 280 nm using spectrophotometer.

Ascorbic acid content was measured by following the method of Kampfenkel et al. (1995). About 0.1 g of leaf samples was extracted with 0.5 ml of 6% trichloroacetic acid (TCA) and centrifuged at 12,000 *g* for 10 min at 4°C. This assay was based on the reduction of ferric ion (Fe^3+^) to ferrous ion (Fe^2+^) with AsA in acid solution, followed by formation of a red chelate between Fe^2+^ and 2,2′-dipyridyl. Samples were finally read for absorbance at 525 nm using spectrophotometer.

Malondialdehyde content was performed using the thiobarbituric acid method (Heath and Packer, 1968). The supernatant (1 ml in volume) was mixed with 1 ml of thiobarbituric acid (0.6%) and then maintained in boiling water bath for 15 min. After cooling, the mixture was centrifuged at 4,000 *g* for 10 min. The absorbance of supernatant was then determined at 450, 532, and 600 nm, respectively.

### Statistics and Analysis

All the data were analyzed using the Student’s *t*-test with SPSS version 10.0 software (SPSS Incorporation, Chicago, IL, United States). Figures were plotted using GraphPad Prism version 9.00 (GraphPad Company, San Diego, CA, United States).

## Results

### Symptom of Powdery Mildew and Morphological Observation

**FIGURE 1:**
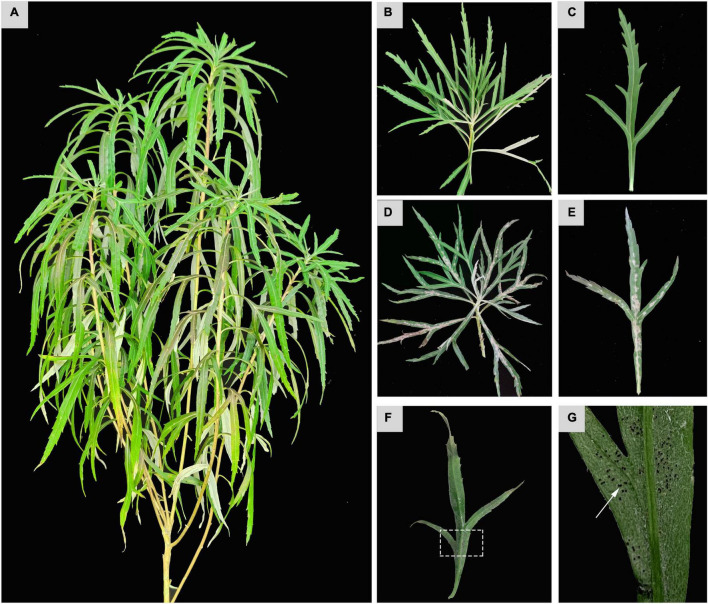
Phenotypic observation of *Golovinomyces artemisiae* infection on *Artemisia selengensis*. Healthy *A. selengensis* **(A)** and partially enlarged images **(B,C)**. *G. artemisiae*-infected leaves **(D,E)** and chasmothecia on the surface **(F,G)**. Those areas indicated by the white dashed square and arrow represents the chasmothecia.

Leaves of *A. selengensis* were major infected parts of the plant for PM (Figure 1D). Whitish colonies with abundant spores were observed on both the adaxial and abaxial surfaces of the infected leaves (Figures 1A–E). Gradually, these infected leaves turned yellow and dark brown with spherical chasmothecia formed on the surfaces (Figures 1F,G).

**FIGURE 2:**
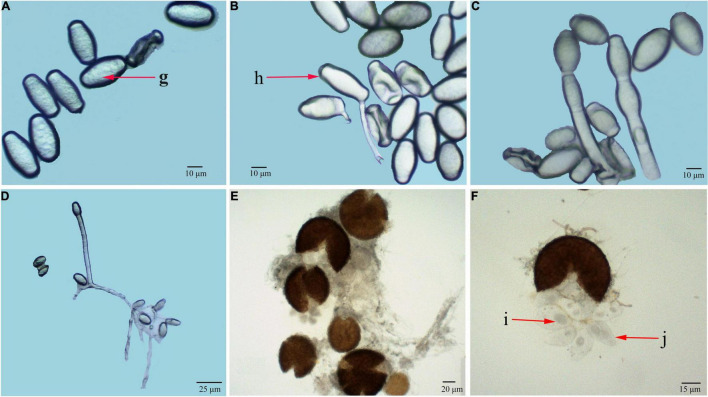
Light microscopic observation of *G. artemisiae* on *A. selengensis*. Conidia **(A,B)**, conidiophore **(C,D)**, and chasmothecia **(E,F)** are shown. Conidia with no fibrosin body (g), conidial germination (h), ascus (i), and ascospores (j) are indicated by the red arrow.

**FIGURE 3:**
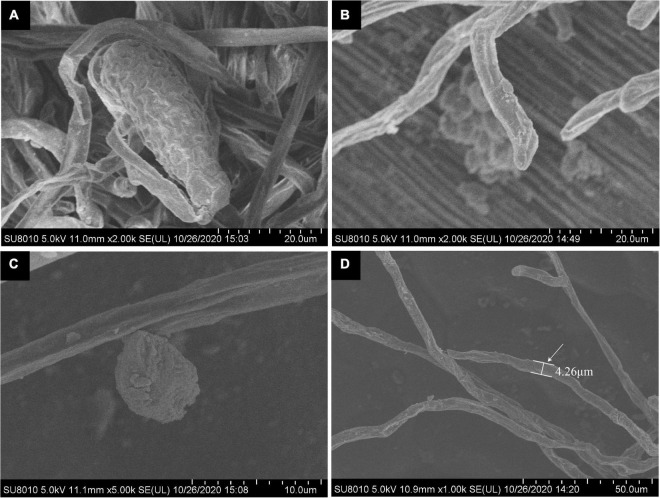
Scanning electron microscopic (SEM) observation of *G. artemisiae* on *A. selengensis*. Conidia **(A)**, hyphae **(B,D)**, and hyphal appressoria **(C)**. The diameter of a hyphae is indicated by the white arrow.

Under optical microscopic observations, conidiophores were measured 98.3 to 132.8 μm in length (average 125.6 μm, *n* = 50) (Figures 2C,D, 3A). Foot cells were straight and approximately grew at right angles from the vegetative hyphae (Figure 2D). Conidia produced in chains were ellipsoidal to ovoid with no fibrosin bodies, measured 28.4 to 40.3 × 12.1 to 20.3 μm (average 31.2 μm × 14.6 μm, *n* = 50) (Figures 2A,B). The chasmothecia were measured 68.3 to 98.1 μm in diameter (average 86.4 μm, *n* = 30) with simple or irregularly branched myceloid appendages formed (Figure 2E). There were 5 to 20 (average 16, *n* = 50) ascus (Figures 2i) observed in ascocarp (Figures 2j), measured 29.1 to 50.4 × 45.2 to 70.1 μm (average 32.1 μm × 56.8 μm, *n* = 50) (Figure 2F). The ascospores (mostly two per ascus) ranging in size from 20.8 to 34.3 × 15.8 to 20.4 μm (average 26.2 μm × 18.1 μm, *n* = 50) were one-celled without fibrosin bodies.

Under scanning electron microscopy, hyphae were hyaline, branched, and sized 2.1 to 8.7 μm (average 5.2 μm) in diameter, while attached appressorium was 6.3 to 9.6 μm (average 7.3 μm) in diameter (Figures 3C,D). The ends of the hyphae had finger-like branches being cylindrical, straight, or slightly curved in shape and measured 14.5 to 24.8 × 6.2 to 12.9 μm (average 20.4 μm × 10.2 μm, *n* = 30) (Figures 3B,D). The abovementioned morphological characteristics of the pathogen were of the typical one of the genus of *Golovinomyces*.

### Molecular Phylogenetic Identification of *Golovinomyces artemisiae*

**FIGURE 4:**
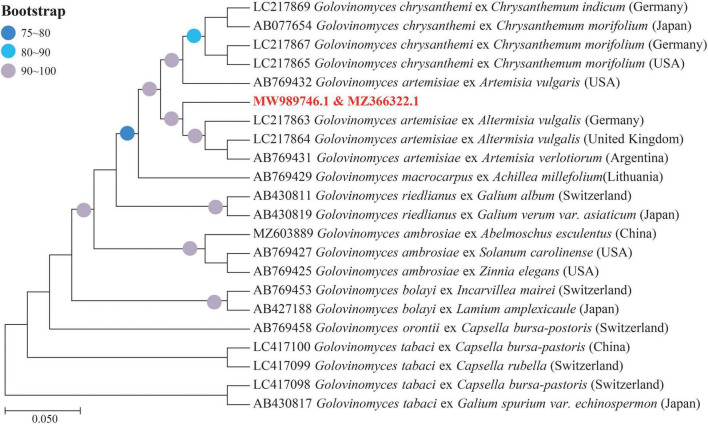
Phylogenetic tree based on the internal transcribed spacer (ITS) and 28S ribosomal DNA (rDNA) sequences of *Golovinomyces* spp. derived from different plant species shows the relationship among strains of several powdery mildew (PM) species, by means of the maximum likelihood (ML) method. Bootstrap values based on 1,000 replications (≥75%) are shown above branches. The identified sequences in this study are indicated by the text in bold, red font color.

Determined ITS and 28S rDNA region of this pathogen being 594 and 860 bp were submitted to GenBank (ITS: MZ366322, 28 rDNA: MW989746). Results of the phylogenetic tree constructed by the ML method showed that this pathogen and *G. artemisiae* belong to the same branch (95% bootstrap support), which was later confirmed by the molecular biosis (Figure 4).

### Pathogenicity Identification of *Golovinomyces artemisiae*

After 8–10 days, the mock-inoculated (CK) leaves remained free of symptoms during the entire period of the experiment in the greenhouse (Supplementary Figure 1A). GI leaves showed typical symptoms, which were consistent with the diseased leaves in field (Supplementary Figure 1B). The experiment was repeated for a few times, which all produced the same results. ITS and 28S rDNA sequences of conidia from the infected leaves further validated the results of the purified *G*. *artemisiae*.

### Leaf Chlorophyll Fluorescence Performances

**FIGURE 5:**
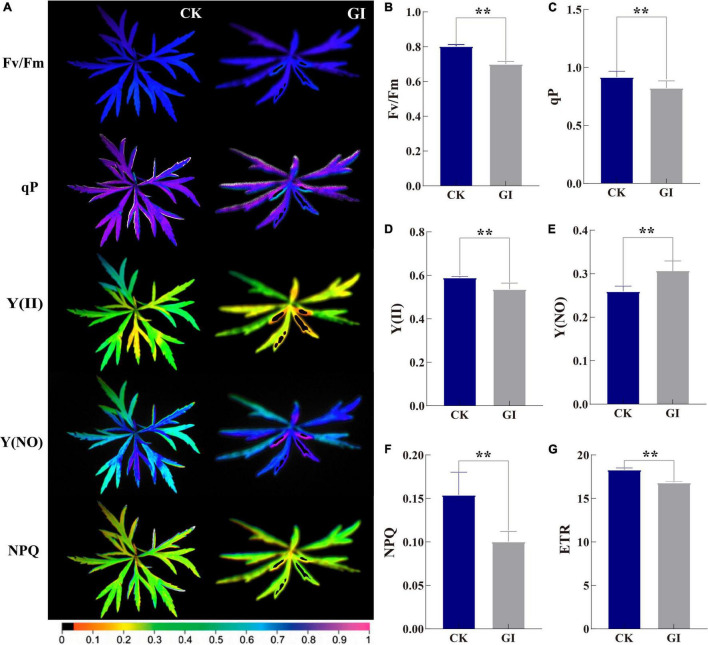
Chlorophyll fluorescence imaging of *G. artemisiae* on *A. selengensis*. Images **(A)** and data of **(B)** maximal quantum yield of PSII photochemistry (Fv/Fm), **(C)** photochemical quenching (qP), **(D)** effective PSII quantum yield [Y(II)], **(E)** quantum yield of non-regulated energy dissipation [Y(NO)], **(F)** non-photochemical quenching (NPQ) coefficient, and **(G)** electron transport rate (ETR) following infection by *G. artemisiae*. Mock-infected control leaf is shown for comparisons. The color scale at the bottom **(A)** indicates values from 0 (black) to 1 (pink). Values are means ± SE of three biological replicates **(B–G)**. Significant differences were calculated using the unpaired Student’s *t*-test (**P ≤ 0.01).

Chlorophyll fluorescence information indicated that the Fv/Fm in CK was significantly greater than that in GI. The images of ChlF parameters showed the emergence of local necrosis in GI. At the same time, the photochemical activity was inhibited and photodamage was occurred (Figure 5A). The value of Fv/Fm for CK was between 0.80 and 0.81 and the value for GI was below 0.80 (Figure 5B). In terms of parameters related to plant light energy absorption and electron transfer, the values of qP, Y(II), and ETR in CK were 11.4, 10.0, and 8.8% higher than those in GI, respectively (Figures 5C,D,G). Obviously, the occurrence of PM inhibited the photosynthetic capacity in *A. selengensis*. Some ChlF parameters associated with light energy consumption showed the opposite expression trends of NPQ and Y(NO) in the two comparison groups. The value of Y(NO) was 4.8% higher in GI than in the CK and NPQ in CK was 53.5%, significantly higher than that in GI (Figures 5E,F).

### Callose, Lignin, and Antioxidant System

**FIGURE 6:**
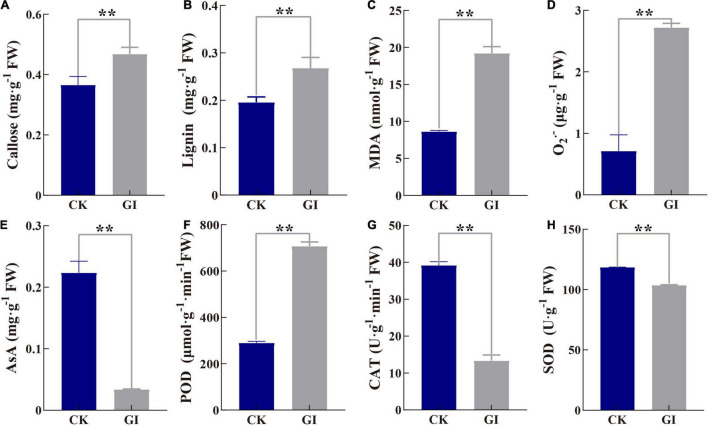
Effects of *G. artemisiae* infection on callose, lignin, and antioxidant-related indexes in *A. selengensis*. **(A)** callose, **(B)** lignin, **(C)** malondialdehyde (MDA), **(D)** superoxide anion (O_2_^–^), **(E)** ascorbic acid (AsA), **(F)** peroxidase (POD), **(G)** catalase (CAT), and **(H)** superoxide dismutase (SOD). Values are means ± SE of three biological replicates. Significant differences were calculated using the unpaired Student’s *t*-test (**P ≤ 0.01).

The contents of callose and lignin were significantly increased to 28.0 and 36.9% in GI compared to CK, respectively (Figures 6A,B). MDA content in GI was higher (1.2-fold) than that in CK (Figure 6C), meanwhile, O_2_^–^ content in GI was significantly higher (2.8-fold) (Figure 6D). In terms of changes in antioxidant enzyme activity, *G. artemisiae* infection resulted in a reduction of 65.9 and 12.6% of CAT and SOD activities in GI, respectively, compared with CK (Figures 6G,H). While AsA, as a non-enzymatic antioxidant, was 84.8% in GI, it is significantly lower than that in CK (Figure 6E). POD activity was 143.9% higher in GI relative to CK (Figure 6F).

## Discussion

Powdery mildew is one of the most frequently occurred fungal diseases in plants around the world. Considerable efforts and investments have been put for the control of the disease via application of proper fungicides and/or breeding of plant varieties tolerant/resistant to the disease. PM appears to be more diverse and the biology of its pathogen seems to be very complex (Glawe, 2008). A holistic approach of combined studies in morphology and analyses of ITS and 28S rDNA regions can accurately identify its causal fungi at the species level (Cunnington et al., 2003). To the best of our knowledge, the *G. artemisiae* cluster comprises sequences obtained from PM hosts of the genera *Artemisia*, *Chrysanthemum*, and *Nipponanthemum* (Bradshaw et al., 2017). In this study, we observed typical symptoms of PM on *A. selengensis* (Figure 1). These symptoms were identical to those previously reported in *A. annua* in Korea (Choi et al., 2014). However, due to specific geographical and climatic environments in Northeast China, physiological race(s) of *G. artemisiae* infecting *A. selengensis* appear to be quite different from those in other regions. Life cycles of PM pathogens can involve both a sexual state (teleomorph) and asexual state (anamorph) or either can be lacking (Glawe, 2008). For example, chasmothecia of *Erysiphe berberidis* DC. were observed in Europe, but they were unknown in western Washington (Glawe, 2008). In this study, chasmothecia were observed, length of conidiophores was less, and pathogenicity was prolonged than that in Korea (Choi et al., 2014). Meanwhile, ITS sequence analysis reflected obvious base mutations (Choi et al., 2014; Chen et al., 2021). Based on the morphology identification and molecular phylogenetic analysis, this study suggested that the pathogen causing PM on *A. selengensis* in both the field and glasshouse in Northeast China is *G. artemisiae*. As the most basic and important indicators of diseases, comprehensive analysis of antioxidant system and photosynthesis indicators is crucial to reveal the phenotype and physiological changes of *A. selengensis* infection with PM.

As one of the most important physiological processes in plants, photosynthesis is inhibited by diseases and other stresses (Durian et al., 2016). Fv/Fm parameter is shown to be a sensitive indicator of photosynthetic performance, with optimal values being close to 0.8 for most plant species (Krause and Weis, 1991). The Fv/Fm values obtained in GI were less than 0.8, indicating the damage to the photosynthetic apparatus due to *G. artemisiae* infection (Figure 5B). Moreover, ETR was inhibited by PM in GI, leading to further reduction in the degree of openness of PS II reaction center (Figure 5G). qP was decreased in GI, which was consistent with the decreasing trend in leaves of *Brassica juncea* with a mosaic virus infection (Guo et al., 2005). The accumulation of reactive intermediates is prevented by increasing the NPQ level in bean (*Phaseolus vulgaris*), which dissipates excess light energy absorbed by the light-harvesting complex harmlessly (Muller et al., 2001; Tietz et al., 2017). Therefore, the progressively increased Y(NO) values and decreased NPQ values indicated the photooxidative damage in GI (Figures 5E,F). It can be further inferred from those Y(II) value that PM caused a decreased energy used for photochemical reactions in GI (Figure 5B), highlighting the reduction of the photosynthetic rate in *A. selengensis* following *G. artemisiae* infection. Early detection of wheat leaves with PM infection by means of fluorescence imaging was 2–3 days before visual symptoms became apparent (Kuckenberg et al., 2009). In this study, the ChlF imaging exhibited the parts of GI leaves infected by PM was different from the surrounding area. The health status of *A. selengensis* can be determined by monitoring the change of Fv/Fm value. Collectively, ChlF is essential for detecting PM epidemics, examining plant health in a timely manner without causing damage.

Plants respond to pathogen invasion by activating a series of defense responses. The deposition of callose after *Colletotrichum gloeosporioides* inoculation of *Stylosanthes guianensis* was associated with cultivar resistance (Sharp et al., 1990). Our results showed that the damage degree of *G. artemisiae* by PM may be mitigated by the increase of callose content in GI (Figure 6A). The increase of lignin content enhanced the activity of POD, which was consistent with the results in *Arabidopsis* (Lee et al., 2018). The synergistic effect of increased lignin content and enhanced POD activity enhanced the resistance of *A. selengensis* to PM (Figures 6B,F). However, in different mustard (*B. juncea* L.) cultivars, the lignin content of *Erysiphe polygoni* DC. in the preinfected stage was higher than that in the diseased stage (Rathod and Chatrabhuji, 2010). Although numerous studies have shown that POD activity is positively correlated with plant disease resistance (Zhang et al., 2020), POD activity in susceptible cultivars is higher than that in resistant cultivars of pumpkin kernel (*Cucurbita pepo* L.) (Zhang et al., 2020, 2021). Thus, the most obviously increased POD activity acted essentially in the hydrolysis of H_2_O_2_ in GI (Figure 6F). These results exhibited great difference changes of relevant indexes after the occurrence of diseases in different plant species.

Reactive oxygen species production is one of the earliest cellular responses following successful pathogen recognition (Sharma et al., 2012; Camejo et al., 2019). O_2_^–^ or H_2_O_2_ generation in apoplast of *Arabidopsis* was infected by *P. syringae* (Grant et al., 2000). In this study, O_2_^–^ content increased by about threefold in GI compared to CK, indicating a serious damage in *A. selengensis* caused by *G. artemisiae* infection (Figure 6D). As another toxic byproduct of ROS metabolism, MDA significantly increased in GI, which was consistent with that in roots of brittle leaf disease-affected date palm (*Phoenix dactylifera* L.) (Saidi et al., 2012). Increased SOD activity has been pinpointed as the key ROS scavenger in response to *Erwinia amylovora* in pear (*Pyrus communis* L.) (Azarabadi et al., 2017). However, a higher potential of CAT activity leads to lower H_2_O_2_ accumulation in rice infected with *M. oryzae* (Hou et al., 2015). Our results showed that the antioxidant capacity was limited due to significantly decreased CAT and SOD enzymes activities in GI (Figures 6G,H). AsA accumulation triggers defense system response in cacao (*Theobroma cacao*) tissues infected by *Moniliophthora perniciosa* (Dias et al., 2011). Moreover, the suppression of AsA synthesis affects the photosynthetic electron transport in tomato infected with *P. syringae* (Yang et al., 2017). In this study, the decreasing AsA content inhibited disease resistance and photosynthesis in GI (Figure 6E). Previous study showed that inhabitation in photosynthetic electron transport inevitably led to the formation of O_2_^–^ in wheat invaded by pathogens (Yang and Luo, 2021). The levels of antioxidative systems and antioxidants were further increased (Yang and Luo, 2021). Combined with the decreased ETR and significantly increased O_2_^–^ in GI, we speculate that photosynthesis should be affected by fungus earlier than the antioxidant system. In conclusion, the pathogen on *A. selengensis* leaves with typical PM characteristics was purified. The conidia, conidiophore, and hyphae of the pathogen were observed under light microscope and SEM. In light of the combined data and information of ITS and 28S rDNA sequence, the PM pathogen of *A. selengensis* was identified as *G. artemisiae*. GI results in damage to photosynthesis in *A. selengensis*. ETR, NPQ, qP, and Y(II) significantly decreased, but Y(NO) increased in infected leaves, further reflecting severe photodamage. Fv/Fm value could be used as the indicator to monitor the health status of *A. selengensis*. In addition, severe stress was reflected due to significant increase in MDA and O_2_^–^ contents in the infected leaves. SOD, CAT activity, and AsA content in GI decreased significantly, with an imbalanced antioxidant system and decreased defense response capacity, while POD activity and lignin contents increased significantly in GI, which are considered to be the key indicators against *G. artemisiae*. The results may help to design PM control approaches for integrating disease control in *A. selengensis* and likewise plants.

## Data Availability Statement

The datasets presented in this study can be found in online repositories. The names of the repository/repositories and accession number(s) can be found below: https://www.ncbi.nlm.nih.gov/genbank/ (ITS: MZ366322, 28 rDNA: MW989746).

## Author Contributions

ZG and XS performed the experiment and data analysis and drafted the manuscript. LD, LX, and LQ helped in collection of data of the experiment. FX contributed to data interpretation and manuscript writing. DQ and YC designed and supervised the experiment. All authors agreed to submit the manuscript for publication.

## Conflict of Interest

The authors declare that the research was conducted in the absence of any commercial or financial relationships that could be construed as a potential conflict of interest.

## Publisher’s Note

All claims expressed in this article are solely those of the authors and do not necessarily represent those of their affiliated organizations, or those of the publisher, the editors and the reviewers. Any product that may be evaluated in this article, or claim that may be made by its manufacturer, is not guaranteed or endorsed by the publisher.

## Footnotes

1
http://www.ncbi.nlm.nih.gov/BLAST
