# Supplementary figures and images for: Identification of Golovinomyces artemisiae Causing Powdery Mildew, Changes in Chlorophyll Fluorescence Parameters, and Antioxidant Levels in Artemisia selengensis
Source: Front Plant Sci. 2022 May 26;13:876050. doi: 10.3389/fpls.2022.876050 (PMC9204253; doi:10.3389/fpls.2022.876050)

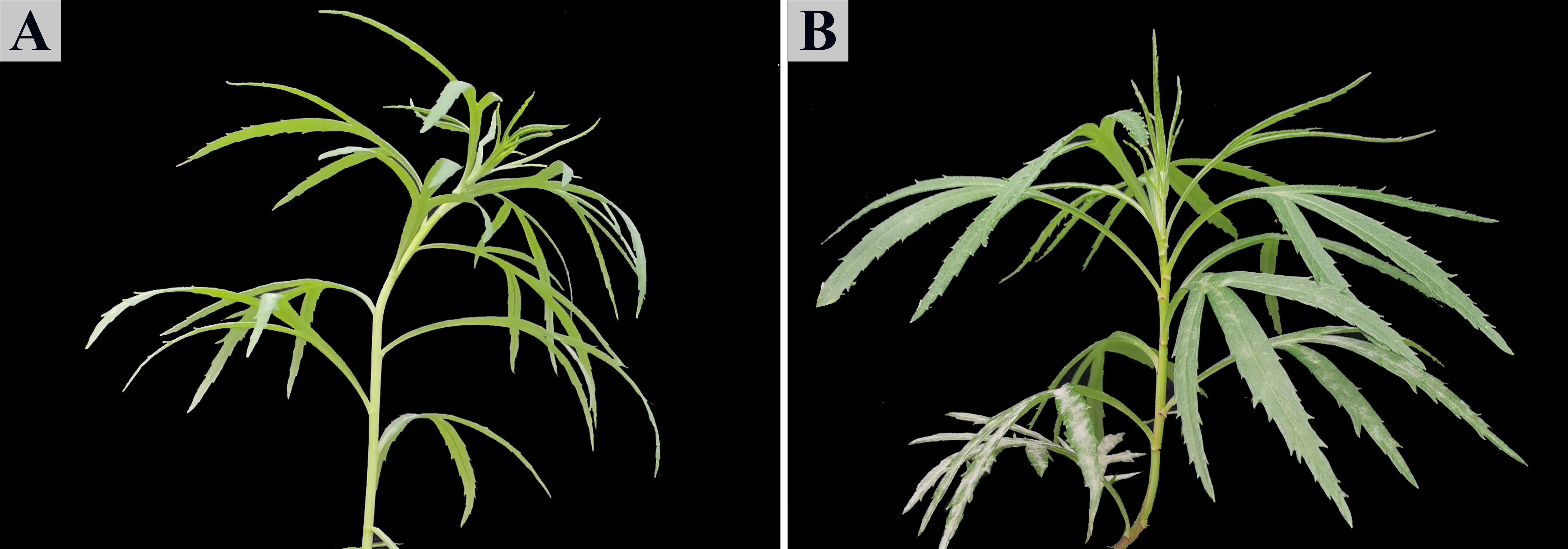

Supplement: Supplementary file 1 [file Image_1.JPEG]
